# 3β,11α-Dihy­droxy-17a-oxa-d-homoandrost-5-en-17-one

**DOI:** 10.1107/S1600536810026516

**Published:** 2010-07-14

**Authors:** Alina Świzdor, Agata Białońska, Teresa Kołek, Anna Panek

**Affiliations:** a Department of Chemistry, Wrocław University of Environmental and Life Sciences, 25. Norwida, 50-375 Wrocław, Poland; b Faculty of Chemistry, University of Wrocław, 14. F. Joliot-Curie, 50-383 Wrocław, Poland

## Abstract

The title compound, C_19_H_28_O_4_, was prepared from DHEA (dehydro­epiandrosterone) by its biotransformation using whole cells of the filamentous fungus *Beauveria bassiana*. The asymmetric unit contains two mol­ecules. The lactone ring is *trans*-positioned to the neighboring six-membered ring. In the crystal structure, O—H⋯O hydrogen bonds form layers, which are linked to each other by O—H⋯O and C—H⋯O hydrogen bonds.

## Related literature

For background information on steroidal lactones and their properties, see: Braunstein (1999 ▶); Brodie & Njar (1998 ▶); Bydal *et al.* (2009 ▶); Feuillan *et al.* (1999 ▶); Li & Parish (1996 ▶); Dunkel (2006 ▶); Penov Gaši *et al.* (2001 ▶, 2005 ▶). For the general method of preparation of the title compound, see: Kołek *et al.* (2008 ▶).
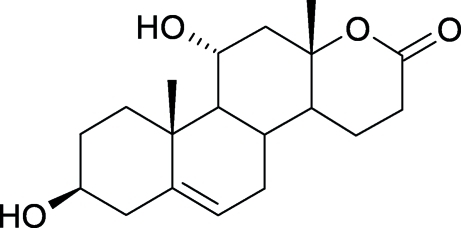


## Experimental

### 

#### Crystal data

C_19_H_28_O_4_
*M* *_r_* = 320.41
Monoclinic, 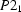

*a* = 11.915 (3) Å
*b* = 9.854 (2) Å
*c* = 14.246 (3) Å
β = 102.66 (3)°
*V* = 1632.0 (6) Å^3^
*Z* = 4
Mo *K*α radiation
μ = 0.09 mm^−1^
*T* = 100 K
0.12 × 0.08 × 0.07 mm

#### Data collection

Kuma KM-4 CCD diffractometer
8286 measured reflections
2921 independent reflections
2062 reflections with *I* > 2σ(*I*)
*R*_int_ = 0.118

#### Refinement

*R*[*F* ^2^ > 2σ(*F* ^2^)] = 0.092
*wR*(*F* ^2^) = 0.167
*S* = 1.13
2921 reflections
415 parameters
1 restraint
H-atom parameters constrained
Δρ_max_ = 0.26 e Å^−3^
Δρ_min_ = −0.28 e Å^−3^

### 

Data collection: *CrysAlis CCD* (Oxford Diffraction, 2009 ▶); cell refinement: *CrysAlis RED* (Oxford Diffraction, 2009 ▶); data reduction: *CrysAlis RED*; program(s) used to solve structure: *SHELXS97* (Sheldrick, 2008 ▶); program(s) used to refine structure: *SHELXL97* (Sheldrick, 2008 ▶); molecular graphics: *XP* (Bruker, 1999 ▶); software used to prepare material for publication: *SHELXL97*.

## Supplementary Material

Crystal structure: contains datablocks global, I. DOI: 10.1107/S1600536810026516/wn2398sup1.cif

Structure factors: contains datablocks I. DOI: 10.1107/S1600536810026516/wn2398Isup2.hkl

Additional supplementary materials:  crystallographic information; 3D view; checkCIF report

## Figures and Tables

**Table 1:** Hydrogen-bond geometry (Å, °)

*D*—H⋯*A*	*D*—H	H⋯*A*	*D*⋯*A*	*D*—H⋯*A*
O3—H31⋯O17*A*^i^	0.84	2.04	2.773 (8)	145
O11—H111⋯O17^ii^	0.84	2.02	2.861 (8)	180
O3*A*—H31*A*⋯O11*A*^iii^	0.84	2.14	2.970 (8)	170
O11*A*—H112⋯O3^iv^	0.91	2.01	2.918 (8)	179
C16—H16*B*⋯O3^v^	0.99	2.47	3.418 (10)	161
C18*A*—H18*E*⋯O3*A*^v^	0.98	2.48	3.443 (9)	167

Symmetry codes: (i) 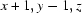; (ii) 
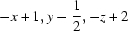
; (iii) 
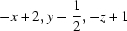
; (iv) 
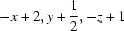
; (v) 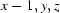.

**Fig. 1.**
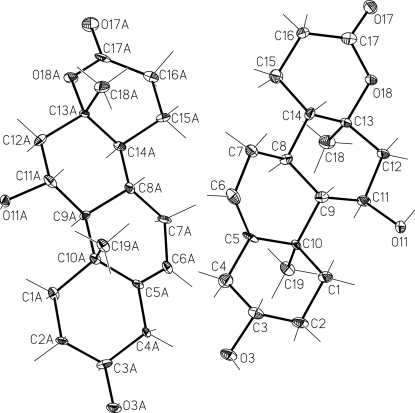
A view of the two crystallographically unrelated 3β,11α-dihydroxy-17a-oxa-D-homo-androst-5-en-17-one molecules together with the numbering scheme employed (30% probability displacement parameters).

**Fig. 2.**
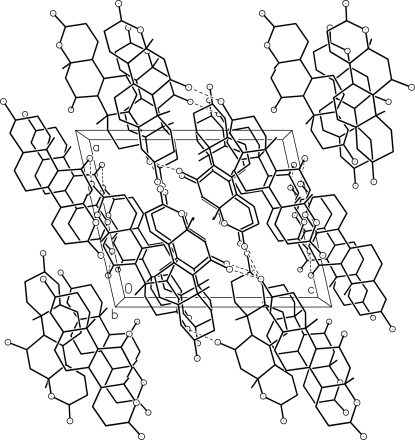
Molecular packing of the title compound, viewed along the [010] direction. Hydrogen atoms are omitted for clarity. Dashed lines indicate hydrogen bonds.

## supplementary crystallographic information

### Comment

A number of steroidal compounds possess anti-aromatase activity and thereby may contribute to the prevention of estrogen-dependent tumors, such as breast cancer (Brodie & Njar, 1998), endometrial cancer (Bydal *et al.*, 2009), prostatic hyperplasia and prostate cancer (Li & Parish, 1996). Among these structures, D-homo lactones of the androstane series represent compounds with promising potency towards human aromatase inhibition (Penov Gaši *et al.*, 2001, 2005). Testolactone (17*a*-oxa-D-homo-androsta-1,4-diene-3,17-dione) is used as a pharmaceutical agent in disorders caused by imbalance between estrogen and androgen action, *e.g.* gynecomastia (Braunstein, 1999) or precocious puberty (Feuillan *et al.*, 1999; Dunkel, 2006).

As a part of our ongoing investigation of the biosynthesis of steroidal lactones, the title compound was obtained.

There are two molecules of the title compound in the asymmetric unit (Fig. 1). O11—H11···O17(-*x* + 1, *y* - 1/2, -*z* + 2) hydrogen bonds are found, also O3A—H31A···O11*a*(-*x* + 2, *y* - 1/2, -*z* + 1) hydrogen bonds, resulting in chains extended along the [010] direction (Table 1). The hydroxyl O11A and O3(-*x* + 2, *y* + 1/2, -*z* + 1) atoms of neighboring chains form hydrogen bonds resulting in layers extended along the [101] direction (Fig. 2). Consecutive layers are linked to each other by O—H···O and C—H···O hydrogen bonds. The hydroxyl O3 and the carbonyl O17A(*x* + 1, *y* - 1, *z*) atoms participate in the O—H···O hydrogen bonds as donor and acceptor, respectively. In the C—H···O interactions the C16 and C18A atoms are donors, and the O3(*x* - 1, *y*, *z*) and O3A(*x* - 1, *y*, *z*) atoms are their acceptors, respectively.

### Experimental

The title compound was prepared from DHEA (dehydroepiandrosterone) by its biotransformation using whole-cells of filamentous fungus *Beauveria bassiana*, according to the general procedure described in (Kołek *et al.*, 2008). The crude extract of reaction products was subjected to silica gel column chromatography using a mixture of ethyl acetate/dichloromethane/acetone/2-propanol (3:1:0.5:0.15) as eluent. The product of this biotransformation was identified by interpretation of its IR, ^1^H NMR and ^13^C NMR spectroscopic  data. ^1^H-NMR: δ (p.p.m.) in CD_3_OD: 1.20 (s, 19-H3); 1.38 (s, 18-H3); 3.45 (m, 3α-H); 3.88 (m, 11β-H); 5.46 (d, J = 5.7 Hz); ^13^C-NMR: δ (p.p.m.) in CD3OD: 174.4 (C-17); 142.7 (C-5); 121.4 (C-6); 83.6 (C-13); 72.5 (C-3); 69.0 (C-11); 56.9 (C-9); 50.3 (C-12); 47.0 (C-14); 43.3 (C-4); 40.4 (C-1); 39.5 (C-10), 35.5 (C-2); 32.3 (C-7); 32.2 (C-8); 29.4 (C-16); 21.1 (C-18); 20.8 (C-15); 19.2 (C-19); IR ν_max_ (cm^-1^): 3440, 1716. Single crystals of the title compound, suitable for X-ray diffraction analysis, were obtained by slow evaporation from methanol after two weeks at room temperature.

### Refinement

All H atoms bonded to O atoms were located in a difference map and then placed in idealised calculated positions with O—H distances of 0.84 Å. All H atoms bonded to C atoms were placed in calculated positions with C—H distances of 0.95 - 1.00 Å. All H atoms were refined as riding. In the absence of significant anomalous scattering, Friedel pairs were merged. The absolute configuration of the title compound was assigned on the basis of the known absolute configuration of particular substrates (commercially available)

### Crystal data

**Table:**

C_19_H_28_O_4_	*F*(000) = 696
*M**_r_* = 320.41	*D*_x_ = 1.304 Mg m^−^^3^
Monoclinic, *P*2_1_	Mo *K*α radiation, λ = 0.71073 Å
Hall symbol: P 2yb	Cell parameters from 1957 reflections
*a* = 11.915 (3) Å	θ = 3.1–28.6°
*b* = 9.854 (2) Å	µ = 0.09 mm^−^^1^
*c* = 14.246 (3) Å	*T* = 100 K
β = 102.66 (3)°	Needle, colorless
*V* = 1632.0 (6) Å^3^	0.12 × 0.08 × 0.07 mm
*Z* = 4	

### Data collection

**Table:**

Kuma KM-4 CCD diffractometer	2062 reflections with *I* > 2σ(*I*)
Radiation source: fine-focus sealed tube	*R*_int_ = 0.118
graphite	θ_max_ = 25.0°, θ_min_ = 3.1°
ω scan	*h* = −13→14
8286 measured reflections	*k* = −11→11
2921 independent reflections	*l* = −16→16

### Refinement

**Table:**

Refinement on *F*^2^	Primary atom site location: structure-invariant direct methods
Least-squares matrix: full	Secondary atom site location: difference Fourier map
*R*[*F*^2^ > 2σ(*F*^2^)] = 0.092	Hydrogen site location: inferred from neighbouring sites
*wR*(*F*^2^) = 0.167	H-atom parameters constrained
*S* = 1.13	*w* = 1/[σ^2^(*F*_o_^2^) + (0.0609*P*)^2^] where *P* = (*F*_o_^2^ + 2*F*_c_^2^)/3
2921 reflections	(Δ/σ)_max_ = 0.002
415 parameters	Δρ_max_ = 0.26 e Å^−^^3^
1 restraint	Δρ_min_ = −0.28 e Å^−^^3^

### Special details

**Table:**

Geometry. All e.s.d.'s (except the e.s.d. in the dihedral angle between two l.s. planes) are estimated using the full covariance matrix. The cell e.s.d.'s are taken into account individually in the estimation of e.s.d.'s in distances, angles and torsion angles; correlations between e.s.d.'s in cell parameters are only used when they are defined by crystal symmetry. An approximate (isotropic) treatment of cell e.s.d.'s is used for estimating e.s.d.'s involving l.s. planes.
Refinement. Refinement of *F*^2^ against ALL reflections. The weighted *R*-factor *wR* and goodness of fit *S* are based on *F*^2^, conventional *R*-factors *R* are based on *F*, with *F* set to zero for negative *F*^2^. The threshold expression of *F*^2^ > σ(*F*^2^) is used only for calculating *R*-factors(gt) *etc*. and is not relevant to the choice of reflections for refinement. *R*-factors based on *F*^2^ are statistically about twice as large as those based on *F*, and *R*- factors based on ALL data will be even larger.

### Fractional atomic coordinates and isotropic or equivalent isotropic displacement parameters (Å^2^)

**Table:**

	*x*	*y*	*z*	*U*_iso_*/*U*_eq_	
C1	0.9668 (6)	−0.0351 (9)	0.8744 (7)	0.027 (2)	
H1A	0.9251	−0.1158	0.8433	0.033*	
H1B	0.9861	−0.0514	0.9446	0.033*	
C2	1.0803 (7)	−0.0189 (9)	0.8385 (7)	0.029 (2)	
H2A	1.1256	0.0572	0.8731	0.035*	
H2B	1.1267	−0.1027	0.8529	0.035*	
C3	1.0566 (7)	0.0076 (9)	0.7341 (7)	0.025 (2)	
H3	1.0206	−0.0751	0.6994	0.031*	
O3	1.1595 (4)	0.0405 (6)	0.7006 (4)	0.0286 (16)	
H31	1.1968	−0.0308	0.6967	0.043*	
C4	0.9758 (7)	0.1260 (9)	0.7066 (7)	0.029 (2)	
H4A	0.9538	0.1320	0.6355	0.035*	
H4B	1.0169	0.2107	0.7306	0.035*	
C5	0.8679 (7)	0.1160 (8)	0.7455 (7)	0.024 (2)	
C6	0.7672 (8)	0.1263 (10)	0.6882 (7)	0.037 (3)	
H6	0.7643	0.1284	0.6210	0.045*	
C7	0.6540 (6)	0.1352 (9)	0.7199 (6)	0.023 (2)	
H7A	0.6109	0.0493	0.7042	0.028*	
H7B	0.6070	0.2097	0.6849	0.028*	
C8	0.6749 (6)	0.1611 (8)	0.8280 (6)	0.0162 (19)	
H8	0.7060	0.2553	0.8404	0.019*	
C9	0.7686 (6)	0.0604 (8)	0.8813 (6)	0.019 (2)	
H9	0.7440	−0.0321	0.8561	0.023*	
C10	0.8868 (6)	0.0897 (8)	0.8531 (6)	0.0157 (19)	
C11	0.7781 (6)	0.0549 (9)	0.9891 (6)	0.023 (2)	
H11	0.8203	0.1382	1.0173	0.027*	
O11	0.8426 (4)	−0.0592 (6)	1.0332 (4)	0.0259 (14)	
H111	0.8013	−0.1290	1.0235	0.039*	
C12	0.6615 (6)	0.0549 (9)	1.0183 (7)	0.024 (2)	
H12A	0.6226	−0.0330	1.0001	0.029*	
H12B	0.6744	0.0643	1.0891	0.029*	
C13	0.5839 (6)	0.1694 (8)	0.9707 (6)	0.020 (2)	
C14	0.5621 (6)	0.1529 (9)	0.8640 (6)	0.020 (2)	
H14	0.5312	0.0591	0.8493	0.024*	
C15	0.4689 (7)	0.2504 (8)	0.8136 (7)	0.027 (2)	
H15A	0.4964	0.3451	0.8248	0.033*	
H15B	0.4515	0.2332	0.7435	0.033*	
C16	0.3611 (6)	0.2308 (9)	0.8522 (6)	0.024 (2)	
H16A	0.3152	0.3152	0.8396	0.028*	
H16B	0.3152	0.1577	0.8143	0.028*	
C17	0.3752 (7)	0.1970 (8)	0.9557 (7)	0.023 (2)	
O17	0.2973 (5)	0.2021 (6)	0.9997 (4)	0.0292 (15)	
C18	0.6319 (7)	0.3083 (9)	1.0075 (7)	0.032 (2)	
H18A	0.5809	0.3798	0.9746	0.048*	
H18B	0.7088	0.3198	0.9946	0.048*	
H18C	0.6366	0.3142	1.0770	0.048*	
O18	0.4780 (4)	0.1528 (5)	1.0074 (4)	0.0214 (14)	
C19	0.9460 (7)	0.2132 (8)	0.9097 (6)	0.025 (2)	
H19A	1.0197	0.2303	0.8920	0.038*	
H19B	0.9595	0.1946	0.9789	0.038*	
H19C	0.8965	0.2932	0.8943	0.038*	
C1A	1.0080 (6)	0.6562 (8)	0.5599 (6)	0.0179 (19)	
H1C	0.9721	0.6734	0.4914	0.022*	
H1D	1.0204	0.7453	0.5927	0.022*	
C2A	1.1250 (6)	0.5893 (8)	0.5655 (6)	0.019 (2)	
H2C	1.1147	0.5052	0.5265	0.023*	
H2D	1.1748	0.6514	0.5382	0.023*	
C3A	1.1829 (6)	0.5553 (9)	0.6679 (6)	0.023 (2)	
H3A	1.1985	0.6426	0.7042	0.027*	
O3A	1.2915 (4)	0.4876 (5)	0.6748 (4)	0.0249 (15)	
H31A	1.2807	0.4129	0.6458	0.037*	
C4A	1.1040 (6)	0.4710 (8)	0.7157 (6)	0.0159 (19)	
H4C	1.0966	0.3790	0.6872	0.019*	
H4D	1.1397	0.4617	0.7850	0.019*	
C5A	0.9847 (6)	0.5318 (7)	0.7051 (6)	0.018 (2)	
C6A	0.9427 (7)	0.5484 (8)	0.7832 (6)	0.022 (2)	
H6A	0.9910	0.5259	0.8435	0.026*	
C7A	0.8240 (6)	0.6003 (8)	0.7828 (6)	0.021 (2)	
H7C	0.8299	0.6927	0.8107	0.025*	
H7D	0.7879	0.5410	0.8240	0.025*	
C8A	0.7465 (6)	0.6047 (8)	0.6807 (6)	0.017 (2)	
H8A	0.7197	0.5105	0.6617	0.021*	
C9A	0.8139 (6)	0.6586 (8)	0.6069 (5)	0.0151 (19)	
H9A	0.8412	0.7519	0.6281	0.018*	
C10A	0.9220 (6)	0.5721 (8)	0.6053 (6)	0.0154 (19)	
O11A	0.7782 (4)	0.7336 (5)	0.4348 (4)	0.0206 (13)	
H112	0.7982	0.6734	0.3932	0.031*	
C11A	0.7297 (6)	0.6732 (8)	0.5095 (6)	0.023 (2)	
H11A	0.6993	0.5812	0.4875	0.028*	
C12A	0.6271 (7)	0.7661 (8)	0.5166 (6)	0.020 (2)	
H12C	0.6559	0.8577	0.5381	0.024*	
H12D	0.5751	0.7749	0.4524	0.024*	
C13A	0.5615 (6)	0.7080 (8)	0.5867 (6)	0.0151 (19)	
C14A	0.6422 (6)	0.6918 (8)	0.6842 (6)	0.0163 (19)	
H14A	0.6714	0.7844	0.7056	0.020*	
C15A	0.5714 (6)	0.6418 (8)	0.7560 (6)	0.019 (2)	
H15C	0.5430	0.5489	0.7381	0.023*	
H15D	0.6216	0.6378	0.8213	0.023*	
C16A	0.4701 (7)	0.7344 (9)	0.7573 (6)	0.026 (2)	
H16C	0.4069	0.6782	0.7715	0.031*	
H16D	0.4933	0.7989	0.8113	0.031*	
C17A	0.4230 (7)	0.8149 (9)	0.6678 (7)	0.026 (2)	
O17A	0.3402 (5)	0.8891 (6)	0.6618 (5)	0.0392 (18)	
C18A	0.4953 (6)	0.5794 (9)	0.5487 (6)	0.027 (2)	
H18D	0.4460	0.5977	0.4853	0.041*	
H18E	0.4477	0.5512	0.5933	0.041*	
H18F	0.5498	0.5070	0.5431	0.041*	
O18A	0.4727 (4)	0.8118 (6)	0.5927 (4)	0.0279 (16)	
C19A	0.8888 (7)	0.4390 (8)	0.5463 (6)	0.021 (2)	
H19D	0.8344	0.3868	0.5744	0.031*	
H19E	0.9581	0.3847	0.5479	0.031*	
H19F	0.8531	0.4621	0.4795	0.031*	

### Atomic displacement parameters (Å^2^)

**Table:**

	*U*^11^	*U*^22^	*U*^33^	*U*^12^	*U*^13^	*U*^23^
C1	0.017 (5)	0.029 (5)	0.037 (6)	0.006 (4)	0.008 (4)	0.004 (5)
C2	0.015 (4)	0.028 (5)	0.046 (7)	0.005 (4)	0.013 (4)	−0.002 (5)
C3	0.019 (5)	0.029 (5)	0.030 (6)	−0.003 (4)	0.010 (4)	−0.007 (4)
O3	0.019 (3)	0.030 (4)	0.041 (4)	0.001 (3)	0.016 (3)	−0.005 (3)
C4	0.028 (5)	0.032 (6)	0.028 (6)	0.005 (4)	0.008 (4)	−0.001 (5)
C5	0.016 (4)	0.019 (5)	0.044 (7)	−0.001 (4)	0.022 (4)	−0.006 (4)
C6	0.036 (6)	0.049 (7)	0.033 (7)	−0.001 (5)	0.021 (5)	0.007 (5)
C7	0.022 (5)	0.024 (5)	0.025 (6)	−0.001 (4)	0.009 (4)	0.001 (4)
C8	0.015 (4)	0.017 (4)	0.015 (5)	0.000 (3)	−0.001 (4)	−0.007 (4)
C9	0.018 (4)	0.017 (5)	0.022 (6)	0.003 (4)	0.005 (4)	−0.006 (4)
C10	0.014 (4)	0.012 (4)	0.022 (6)	−0.004 (3)	0.006 (4)	0.004 (4)
C11	0.016 (4)	0.022 (5)	0.033 (6)	0.006 (4)	0.011 (4)	0.001 (4)
O11	0.021 (3)	0.026 (3)	0.031 (4)	0.002 (3)	0.006 (3)	0.014 (3)
C12	0.014 (4)	0.028 (5)	0.033 (6)	0.005 (4)	0.010 (4)	0.009 (4)
C13	0.012 (4)	0.024 (5)	0.026 (6)	0.000 (4)	0.007 (4)	0.005 (4)
C14	0.022 (5)	0.023 (5)	0.013 (5)	0.004 (4)	0.003 (4)	0.003 (4)
C15	0.027 (5)	0.017 (4)	0.038 (7)	0.001 (4)	0.009 (4)	0.003 (4)
C16	0.016 (4)	0.031 (5)	0.023 (6)	0.002 (4)	0.002 (4)	0.004 (4)
C17	0.020 (5)	0.016 (4)	0.033 (6)	−0.001 (4)	0.006 (4)	−0.010 (4)
O17	0.027 (3)	0.027 (3)	0.036 (4)	0.001 (3)	0.011 (3)	0.005 (3)
C18	0.027 (5)	0.030 (5)	0.039 (7)	−0.003 (4)	0.005 (5)	−0.003 (5)
O18	0.015 (3)	0.025 (3)	0.027 (4)	0.004 (3)	0.009 (3)	0.005 (3)
C19	0.028 (5)	0.020 (5)	0.030 (6)	−0.005 (4)	0.012 (4)	−0.006 (4)
C1A	0.024 (5)	0.009 (4)	0.024 (5)	0.001 (4)	0.013 (4)	0.000 (4)
C2A	0.008 (4)	0.019 (4)	0.031 (6)	0.002 (3)	0.007 (4)	0.004 (4)
C3A	0.012 (4)	0.032 (5)	0.022 (6)	0.006 (4)	−0.001 (4)	−0.007 (4)
O3A	0.013 (3)	0.025 (3)	0.038 (4)	0.003 (3)	0.010 (3)	0.000 (3)
C4A	0.013 (4)	0.015 (5)	0.018 (5)	−0.007 (4)	0.002 (4)	0.005 (4)
C5A	0.011 (4)	0.005 (4)	0.037 (6)	−0.003 (3)	0.008 (4)	0.004 (4)
C6A	0.022 (5)	0.019 (5)	0.025 (6)	−0.007 (4)	0.008 (4)	0.006 (4)
C7A	0.008 (4)	0.024 (5)	0.032 (6)	−0.003 (4)	0.006 (4)	0.000 (4)
C8A	0.017 (4)	0.009 (4)	0.028 (6)	0.001 (3)	0.009 (4)	0.006 (4)
C9A	0.012 (4)	0.024 (5)	0.009 (5)	0.002 (4)	0.000 (3)	0.000 (4)
C10A	0.016 (4)	0.009 (4)	0.023 (5)	−0.001 (3)	0.007 (4)	−0.001 (4)
O11A	0.022 (3)	0.025 (3)	0.019 (3)	0.007 (3)	0.012 (3)	0.007 (3)
C11A	0.024 (5)	0.017 (5)	0.033 (6)	−0.005 (4)	0.016 (4)	−0.001 (4)
C12A	0.019 (4)	0.021 (5)	0.016 (5)	0.003 (4)	−0.004 (4)	−0.001 (4)
C13A	0.007 (4)	0.015 (4)	0.023 (5)	−0.001 (3)	0.003 (4)	0.005 (4)
C14A	0.013 (4)	0.019 (4)	0.018 (5)	−0.005 (3)	0.005 (4)	−0.005 (4)
C15A	0.011 (4)	0.024 (5)	0.021 (6)	0.002 (4)	−0.002 (4)	−0.005 (4)
C16A	0.017 (4)	0.030 (5)	0.032 (6)	−0.005 (4)	0.008 (4)	−0.004 (5)
C17A	0.011 (4)	0.028 (5)	0.044 (7)	−0.002 (4)	0.015 (4)	−0.002 (5)
O17A	0.030 (4)	0.044 (4)	0.049 (5)	0.019 (3)	0.020 (3)	0.007 (4)
C18A	0.020 (5)	0.033 (5)	0.027 (6)	−0.002 (4)	0.000 (4)	−0.004 (4)
O18A	0.016 (3)	0.028 (4)	0.043 (5)	0.011 (3)	0.014 (3)	0.008 (3)
C19A	0.025 (5)	0.018 (4)	0.023 (5)	−0.001 (4)	0.015 (4)	−0.005 (4)

### Geometric parameters (Å, °)

**Table:**

C1—C10	1.545 (10)	C1A—C2A	1.528 (10)
C1—C2	1.554 (10)	C1A—C10A	1.564 (10)
C1—H1A	0.9900	C1A—H1C	0.9900
C1—H1B	0.9900	C1A—H1D	0.9900
C2—C3	1.474 (12)	C2A—C3A	1.509 (11)
C2—H2A	0.9900	C2A—H2C	0.9900
C2—H2B	0.9900	C2A—H2D	0.9900
C3—O3	1.447 (9)	C3A—O3A	1.440 (8)
C3—C4	1.509 (11)	C3A—C4A	1.522 (10)
C3—H3	1.0000	C3A—H3A	1.0000
O3—H31	0.8400	O3A—H31A	0.8400
C4—C5	1.512 (10)	C4A—C5A	1.519 (10)
C4—H4A	0.9900	C4A—H4C	0.9900
C4—H4B	0.9900	C4A—H4D	0.9900
C5—C6	1.298 (12)	C5A—C6A	1.325 (10)
C5—C10	1.522 (12)	C5A—C10A	1.508 (11)
C6—C7	1.516 (10)	C6A—C7A	1.503 (10)
C6—H6	0.9500	C6A—H6A	0.9500
C7—C8	1.526 (10)	C7A—C8A	1.543 (11)
C7—H7A	0.9900	C7A—H7C	0.9900
C7—H7B	0.9900	C7A—H7D	0.9900
C8—C14	1.542 (10)	C8A—C14A	1.521 (10)
C8—C9	1.560 (10)	C8A—C9A	1.549 (10)
C8—H8	1.0000	C8A—H8A	1.0000
C9—C11	1.516 (11)	C9A—C11A	1.530 (11)
C9—C10	1.574 (9)	C9A—C10A	1.549 (10)
C9—H9	1.0000	C9A—H9A	1.0000
C10—C19	1.543 (10)	C10A—C19A	1.561 (10)
C11—O11	1.427 (9)	O11A—C11A	1.446 (9)
C11—C12	1.535 (10)	O11A—H112	0.9069
C11—H11	1.0000	C11A—C12A	1.549 (10)
O11—H111	0.8400	C11A—H11A	1.0000
C12—C13	1.520 (11)	C12A—C13A	1.510 (10)
C12—H12A	0.9900	C12A—H12C	0.9900
C12—H12B	0.9900	C12A—H12D	0.9900
C13—O18	1.477 (8)	C13A—O18A	1.487 (9)
C13—C14	1.494 (11)	C13A—C14A	1.514 (10)
C13—C18	1.531 (12)	C13A—C18A	1.528 (11)
C14—C15	1.524 (11)	C14A—C15A	1.543 (10)
C14—H14	1.0000	C14A—H14A	1.0000
C15—C16	1.516 (10)	C15A—C16A	1.517 (10)
C15—H15A	0.9900	C15A—H15C	0.9900
C15—H15B	0.9900	C15A—H15D	0.9900
C16—C17	1.484 (11)	C16A—C17A	1.503 (12)
C16—H16A	0.9900	C16A—H16C	0.9900
C16—H16B	0.9900	C16A—H16D	0.9900
C17—O17	1.230 (9)	C17A—O17A	1.216 (9)
C17—O18	1.356 (9)	C17A—O18A	1.331 (10)
C18—H18A	0.9800	C18A—H18D	0.9800
C18—H18B	0.9800	C18A—H18E	0.9800
C18—H18C	0.9800	C18A—H18F	0.9800
C19—H19A	0.9800	C19A—H19D	0.9800
C19—H19B	0.9800	C19A—H19E	0.9800
C19—H19C	0.9800	C19A—H19F	0.9800
			
C10—C1—C2	113.4 (7)	C2A—C1A—C10A	115.0 (7)
C10—C1—H1A	108.9	C2A—C1A—H1C	108.5
C2—C1—H1A	108.9	C10A—C1A—H1C	108.5
C10—C1—H1B	108.9	C2A—C1A—H1D	108.5
C2—C1—H1B	108.9	C10A—C1A—H1D	108.5
H1A—C1—H1B	107.7	H1C—C1A—H1D	107.5
C3—C2—C1	111.2 (7)	C3A—C2A—C1A	111.4 (6)
C3—C2—H2A	109.4	C3A—C2A—H2C	109.3
C1—C2—H2A	109.4	C1A—C2A—H2C	109.3
C3—C2—H2B	109.4	C3A—C2A—H2D	109.3
C1—C2—H2B	109.4	C1A—C2A—H2D	109.3
H2A—C2—H2B	108.0	H2C—C2A—H2D	108.0
O3—C3—C2	112.6 (7)	O3A—C3A—C2A	112.5 (6)
O3—C3—C4	106.2 (6)	O3A—C3A—C4A	110.6 (7)
C2—C3—C4	111.7 (7)	C2A—C3A—C4A	110.9 (6)
O3—C3—H3	108.8	O3A—C3A—H3A	107.6
C2—C3—H3	108.8	C2A—C3A—H3A	107.6
C4—C3—H3	108.8	C4A—C3A—H3A	107.6
C3—O3—H31	109.5	C3A—O3A—H31A	109.5
C3—C4—C5	113.6 (7)	C5A—C4A—C3A	113.4 (6)
C3—C4—H4A	108.9	C5A—C4A—H4C	108.9
C5—C4—H4A	108.9	C3A—C4A—H4C	108.9
C3—C4—H4B	108.9	C5A—C4A—H4D	108.9
C5—C4—H4B	108.9	C3A—C4A—H4D	108.9
H4A—C4—H4B	107.7	H4C—C4A—H4D	107.7
C6—C5—C4	120.4 (8)	C6A—C5A—C10A	124.0 (7)
C6—C5—C10	124.0 (7)	C6A—C5A—C4A	118.9 (8)
C4—C5—C10	115.5 (7)	C10A—C5A—C4A	117.2 (7)
C5—C6—C7	125.2 (9)	C5A—C6A—C7A	124.5 (8)
C5—C6—H6	117.4	C5A—C6A—H6A	117.7
C7—C6—H6	117.4	C7A—C6A—H6A	117.7
C6—C7—C8	110.6 (7)	C6A—C7A—C8A	112.4 (7)
C6—C7—H7A	109.5	C6A—C7A—H7C	109.1
C8—C7—H7A	109.5	C8A—C7A—H7C	109.1
C6—C7—H7B	109.5	C6A—C7A—H7D	109.1
C8—C7—H7B	109.5	C8A—C7A—H7D	109.1
H7A—C7—H7B	108.1	H7C—C7A—H7D	107.9
C7—C8—C14	111.4 (6)	C14A—C8A—C7A	108.1 (6)
C7—C8—C9	109.0 (6)	C14A—C8A—C9A	112.2 (6)
C14—C8—C9	112.8 (6)	C7A—C8A—C9A	111.0 (6)
C7—C8—H8	107.8	C14A—C8A—H8A	108.5
C14—C8—H8	107.8	C7A—C8A—H8A	108.5
C9—C8—H8	107.8	C9A—C8A—H8A	108.5
C11—C9—C8	113.8 (6)	C11A—C9A—C8A	108.3 (6)
C11—C9—C10	113.1 (6)	C11A—C9A—C10A	114.8 (6)
C8—C9—C10	110.1 (6)	C8A—C9A—C10A	112.2 (6)
C11—C9—H9	106.4	C11A—C9A—H9A	107.1
C8—C9—H9	106.4	C8A—C9A—H9A	107.1
C10—C9—H9	106.4	C10A—C9A—H9A	107.1
C5—C10—C19	110.0 (6)	C5A—C10A—C9A	111.9 (6)
C5—C10—C1	106.4 (7)	C5A—C10A—C19A	107.6 (6)
C19—C10—C1	109.5 (6)	C9A—C10A—C19A	110.9 (6)
C5—C10—C9	110.2 (6)	C5A—C10A—C1A	107.2 (6)
C19—C10—C9	110.1 (6)	C9A—C10A—C1A	109.7 (6)
C1—C10—C9	110.7 (6)	C19A—C10A—C1A	109.5 (6)
O11—C11—C9	112.8 (6)	C11A—O11A—H112	114.6
O11—C11—C12	108.2 (6)	O11A—C11A—C9A	114.7 (6)
C9—C11—C12	113.8 (7)	O11A—C11A—C12A	104.6 (6)
O11—C11—H11	107.2	C9A—C11A—C12A	111.4 (6)
C9—C11—H11	107.2	O11A—C11A—H11A	108.6
C12—C11—H11	107.2	C9A—C11A—H11A	108.6
C11—O11—H111	109.5	C12A—C11A—H11A	108.6
C13—C12—C11	112.2 (7)	C13A—C12A—C11A	110.5 (6)
C13—C12—H12A	109.2	C13A—C12A—H12C	109.6
C11—C12—H12A	109.2	C11A—C12A—H12C	109.6
C13—C12—H12B	109.2	C13A—C12A—H12D	109.6
C11—C12—H12B	109.2	C11A—C12A—H12D	109.6
H12A—C12—H12B	107.9	H12C—C12A—H12D	108.1
O18—C13—C14	112.1 (6)	O18A—C13A—C12A	104.8 (6)
O18—C13—C12	104.1 (6)	O18A—C13A—C14A	109.7 (6)
C14—C13—C12	109.1 (7)	C12A—C13A—C14A	109.3 (6)
O18—C13—C18	105.1 (6)	O18A—C13A—C18A	105.8 (6)
C14—C13—C18	114.5 (7)	C12A—C13A—C18A	112.4 (7)
C12—C13—C18	111.5 (7)	C14A—C13A—C18A	114.3 (7)
C13—C14—C15	110.9 (7)	C13A—C14A—C8A	112.9 (6)
C13—C14—C8	111.3 (6)	C13A—C14A—C15A	108.2 (6)
C15—C14—C8	114.1 (6)	C8A—C14A—C15A	114.2 (7)
C13—C14—H14	106.7	C13A—C14A—H14A	107.0
C15—C14—H14	106.7	C8A—C14A—H14A	107.0
C8—C14—H14	106.7	C15A—C14A—H14A	107.0
C16—C15—C14	109.7 (7)	C16A—C15A—C14A	111.6 (7)
C16—C15—H15A	109.7	C16A—C15A—H15C	109.3
C14—C15—H15A	109.7	C14A—C15A—H15C	109.3
C16—C15—H15B	109.7	C16A—C15A—H15D	109.3
C14—C15—H15B	109.7	C14A—C15A—H15D	109.3
H15A—C15—H15B	108.2	H15C—C15A—H15D	108.0
C17—C16—C15	117.9 (7)	C17A—C16A—C15A	117.2 (7)
C17—C16—H16A	107.8	C17A—C16A—H16C	108.0
C15—C16—H16A	107.8	C15A—C16A—H16C	108.0
C17—C16—H16B	107.8	C17A—C16A—H16D	108.0
C15—C16—H16B	107.8	C15A—C16A—H16D	108.0
H16A—C16—H16B	107.2	H16C—C16A—H16D	107.2
O17—C17—O18	115.4 (8)	O17A—C17A—O18A	117.0 (9)
O17—C17—C16	124.3 (7)	O17A—C17A—C16A	121.6 (8)
O18—C17—C16	120.3 (7)	O18A—C17A—C16A	121.4 (7)
C13—C18—H18A	109.5	C13A—C18A—H18D	109.5
C13—C18—H18B	109.5	C13A—C18A—H18E	109.5
H18A—C18—H18B	109.5	H18D—C18A—H18E	109.5
C13—C18—H18C	109.5	C13A—C18A—H18F	109.5
H18A—C18—H18C	109.5	H18D—C18A—H18F	109.5
H18B—C18—H18C	109.5	H18E—C18A—H18F	109.5
C17—O18—C13	120.8 (6)	C17A—O18A—C13A	120.6 (6)
C10—C19—H19A	109.5	C10A—C19A—H19D	109.5
C10—C19—H19B	109.5	C10A—C19A—H19E	109.5
H19A—C19—H19B	109.5	H19D—C19A—H19E	109.5
C10—C19—H19C	109.5	C10A—C19A—H19F	109.5
H19A—C19—H19C	109.5	H19D—C19A—H19F	109.5
H19B—C19—H19C	109.5	H19E—C19A—H19F	109.5
			
C10—C1—C2—C3	−57.8 (10)	C10A—C1A—C2A—C3A	−56.1 (9)
C1—C2—C3—O3	172.5 (6)	C1A—C2A—C3A—O3A	177.9 (7)
C1—C2—C3—C4	53.2 (9)	C1A—C2A—C3A—C4A	53.5 (9)
O3—C3—C4—C5	−173.8 (7)	O3A—C3A—C4A—C5A	−176.0 (7)
C2—C3—C4—C5	−50.7 (10)	C2A—C3A—C4A—C5A	−50.5 (9)
C3—C4—C5—C6	−126.5 (10)	C3A—C4A—C5A—C6A	−128.5 (8)
C3—C4—C5—C10	51.8 (10)	C3A—C4A—C5A—C10A	50.5 (9)
C4—C5—C6—C7	−172.1 (7)	C10A—C5A—C6A—C7A	4.5 (12)
C10—C5—C6—C7	9.7 (15)	C4A—C5A—C6A—C7A	−176.7 (7)
C5—C6—C7—C8	12.2 (12)	C5A—C6A—C7A—C8A	11.9 (11)
C6—C7—C8—C14	−172.9 (7)	C6A—C7A—C8A—C14A	−165.1 (6)
C6—C7—C8—C9	−47.9 (9)	C6A—C7A—C8A—C9A	−41.8 (9)
C7—C8—C9—C11	−167.3 (7)	C14A—C8A—C9A—C11A	−53.8 (8)
C14—C8—C9—C11	−43.0 (9)	C7A—C8A—C9A—C11A	−174.7 (6)
C7—C8—C9—C10	64.6 (8)	C14A—C8A—C9A—C10A	178.5 (7)
C14—C8—C9—C10	−171.2 (7)	C7A—C8A—C9A—C10A	57.5 (8)
C6—C5—C10—C19	−115.1 (9)	C6A—C5A—C10A—C9A	10.6 (10)
C4—C5—C10—C19	66.7 (8)	C4A—C5A—C10A—C9A	−168.3 (7)
C6—C5—C10—C1	126.5 (9)	C6A—C5A—C10A—C19A	−111.4 (8)
C4—C5—C10—C1	−51.7 (8)	C4A—C5A—C10A—C19A	69.7 (8)
C6—C5—C10—C9	6.5 (11)	C6A—C5A—C10A—C1A	130.9 (8)
C4—C5—C10—C9	−171.7 (6)	C4A—C5A—C10A—C1A	−48.0 (8)
C2—C1—C10—C5	54.3 (9)	C11A—C9A—C10A—C5A	−165.2 (6)
C2—C1—C10—C19	−64.5 (9)	C8A—C9A—C10A—C5A	−41.1 (8)
C2—C1—C10—C9	174.0 (7)	C11A—C9A—C10A—C19A	−45.2 (9)
C11—C9—C10—C5	−171.1 (7)	C8A—C9A—C10A—C19A	79.0 (8)
C8—C9—C10—C5	−42.6 (8)	C11A—C9A—C10A—C1A	75.9 (8)
C11—C9—C10—C19	−49.6 (9)	C8A—C9A—C10A—C1A	−159.9 (6)
C8—C9—C10—C19	79.0 (8)	C2A—C1A—C10A—C5A	50.9 (9)
C11—C9—C10—C1	71.6 (9)	C2A—C1A—C10A—C9A	172.6 (7)
C8—C9—C10—C1	−159.9 (7)	C2A—C1A—C10A—C19A	−65.5 (9)
C8—C9—C11—O11	165.7 (6)	C8A—C9A—C11A—O11A	175.0 (6)
C10—C9—C11—O11	−67.7 (8)	C10A—C9A—C11A—O11A	−58.8 (9)
C8—C9—C11—C12	41.9 (9)	C8A—C9A—C11A—C12A	56.3 (8)
C10—C9—C11—C12	168.4 (6)	C10A—C9A—C11A—C12A	−177.5 (6)
O11—C11—C12—C13	−177.2 (7)	O11A—C11A—C12A—C13A	175.2 (6)
C9—C11—C12—C13	−50.9 (10)	C9A—C11A—C12A—C13A	−60.3 (8)
C11—C12—C13—O18	−179.8 (7)	C11A—C12A—C13A—O18A	175.4 (6)
C11—C12—C13—C14	60.3 (9)	C11A—C12A—C13A—C14A	57.9 (8)
C11—C12—C13—C18	−67.0 (9)	C11A—C12A—C13A—C18A	−70.2 (8)
O18—C13—C14—C15	55.6 (8)	O18A—C13A—C14A—C8A	−170.7 (6)
C12—C13—C14—C15	170.3 (6)	C12A—C13A—C14A—C8A	−56.4 (8)
C18—C13—C14—C15	−64.1 (8)	C18A—C13A—C14A—C8A	70.7 (8)
O18—C13—C14—C8	−176.2 (6)	O18A—C13A—C14A—C15A	61.8 (8)
C12—C13—C14—C8	−61.5 (8)	C12A—C13A—C14A—C15A	176.2 (6)
C18—C13—C14—C8	64.1 (8)	C18A—C13A—C14A—C15A	−56.8 (8)
C7—C8—C14—C13	176.3 (7)	C7A—C8A—C14A—C13A	177.9 (6)
C9—C8—C14—C13	53.4 (8)	C9A—C8A—C14A—C13A	55.3 (8)
C7—C8—C14—C15	−57.3 (9)	C7A—C8A—C14A—C15A	−57.9 (8)
C9—C8—C14—C15	179.8 (7)	C9A—C8A—C14A—C15A	179.5 (6)
C13—C14—C15—C16	−55.1 (9)	C13A—C14A—C15A—C16A	−54.4 (8)
C8—C14—C15—C16	178.3 (7)	C8A—C14A—C15A—C16A	178.9 (7)
C14—C15—C16—C17	35.3 (11)	C14A—C15A—C16A—C17A	25.9 (10)
C15—C16—C17—O17	166.5 (8)	C15A—C16A—C17A—O17A	178.2 (8)
C15—C16—C17—O18	−15.8 (11)	C15A—C16A—C17A—O18A	−4.2 (12)
O17—C17—O18—C13	−166.7 (7)	O17A—C17A—O18A—C13A	−169.8 (7)
C16—C17—O18—C13	15.4 (11)	C16A—C17A—O18A—C13A	12.5 (11)
C14—C13—O18—C17	−35.6 (9)	C12A—C13A—O18A—C17A	−159.4 (7)
C12—C13—O18—C17	−153.4 (7)	C14A—C13A—O18A—C17A	−42.2 (9)
C18—C13—O18—C17	89.3 (8)	C18A—C13A—O18A—C17A	81.6 (8)

### Hydrogen-bond geometry (Å, °)

**Table:**

*D*—H···*A*	*D*—H	H···*A*	*D*···*A*	*D*—H···*A*
O3—H31···O17A^i^	0.84	2.04	2.773 (8)	145
O11—H111···O17^ii^	0.84	2.02	2.861 (8)	180
O3A—H31A···O11A^iii^	0.84	2.14	2.970 (8)	170
O11A—H112···O3^iv^	0.91	2.01	2.918 (8)	179
C16—H16B···O3^v^	0.99	2.47	3.418 (10)	161
C18A—H18E···O3A^v^	0.98	2.48	3.443 (9)	167

Symmetry codes: (i) *x*+1, *y*−1, *z*; (ii) −*x*+1, *y*−1/2, −*z*+2; (iii) −*x*+2, *y*−1/2, −*z*+1; (iv) −*x*+2, *y*+1/2, −*z*+1; (v) *x*−1, *y*, *z*.
